# Partial third nerve palsy after Measles Mumps Rubella vaccination

**DOI:** 10.1186/1824-7288-36-59

**Published:** 2010-09-10

**Authors:** Francesca Manzotti, Chiara Menozzi, Maria R Porta, Jelka G Orsoni

**Affiliations:** 1 Department of Cervico-Facial Sciences, Institute of Ophthalmology, University of Parma, Parma, Italy

## Abstract

**Background:**

Measles Mumps Rubella (MMR) vaccination is known to cause some serious adverse events, such as fever, rash, gland inflammation and neurologic disorders. These include third and sixth cranial nerve palsies.

**Results:**

The case reported describes a partial recurrent oculomotor palsy associated with systemic symptoms following MMR vaccination in a healthy young child. The oculomotor palsy did not recover completely during the follow-up.

**Conclusions:**

Most of the times, measles, mumps and rubella cause mild illness and discomfort; but can also have serious or fatal sequelae. MMR vaccination has been proved to be safe and to reduce significantly the number of reported infections due to these viruses. However, significant adverse events can occur and paediatricians and public health operators should be aware of this aspect.

## Partial third nerve palsy after Measles Mumps Rubella vaccination

Sir,

Measles Mumps Rubella (MMR) vaccination is known to cause some serious adverse events. These can include fever, rash, gland inflammation and neurologic disorders like epilepsy, encephalitis, aseptic meningitis and autistic disorders [1,2].

The case reported below describes a partial recurrent oculomotor palsy following MMR vaccination in a healthy young child.

## Case Report

**Figure 1:**
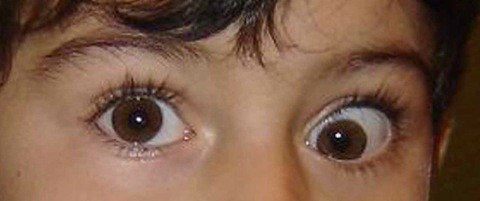
**Deficit of elevation in adduction of the LE**.

A 20-month-old Italian boy received a MMR (MMR VAX PRO^®^) vaccine dose according to the normal vaccination schedule. Twenty days later he presented fever and cutaneous rash, which resolved spontaneously in one day, and left eye (LE) eso-hypotropia, which also lasted 24-48 hrs until complete self-recovery. This episode recurred 20 days later: the ophthalmological examination showed a deficit of elevation in adduction of the LE and was diagnosed as "acquired palsy of the inferior oblique muscle" [Fig. 1]. The remaining neurologic examination was normal. EEG, brain MRI with and without gadolinium, blood count, blood chemistry were normal. Serum titres against main viral agents (Citomegalovirus, Epstein-Barr, Herpes Simplex, Varicella Zoster, Papovavirus B19, Influenza, Parainfluenza, Respiratory Syncytial) were negative, except for a positive IgG titre for Rubella virus. A week later the symptoms partially disappeared without any treatment. The following scheduled neurological controls continued to be normal, while the orthoptic examination showed persisting, but inconstant, weakening of LE inferior oblique muscle function. At the last control, when the child was 5 years old, visual acuity and stereopsis tests were normal, but a mild deficit of the inferior oblique muscle function persisted, with orthophoria in primary position.

## Discussion

MMR viruses are neurotropic. Therefore, although the vaccine is obtained by live attenuated viruses, vaccination may produce neurological disorders. Only a few cases of benign, recurrent cranial nerve palsies, secondary to immunization, have been described. Patients with complete palsy of the third [3] or sixth [4-7] cranial nerve have been reported. In our case, the oculomotor nerve palsy is incomplete i.e. only the inferior oblique muscle is involved in one eye. The previously reported cases resolved spontaneously and completely within several months. In our case, however, a mild deficit of the muscle has persisted several years following vaccination and may be considered permanent.

In conclusion, public confidence in immunization must be maintained because safety and efficacy of MMR vaccination is clearly demonstrated; however potential adverse events must be strictly and carefully monitored.
